# Maternal aerobic running during mid or late gestation improves the quality of oogenesis and folliculogenesis in the ovary of neonatal rats: An experimental study

**DOI:** 10.18502/ijrm.v19i9.9713

**Published:** 2021-10-10

**Authors:** Behpour Yousefi, Raheleh Baradaran, Tamineh Mokhtari, Vahid Semnani, Hamidreza Sameni

**Affiliations:** ^1^Abnormal Uterine Bleeding Research Center, Semnan University of Medical Sciences, Semnan, Iran.; ^2^Department of Anatomy, Medicine Faculty, Semnan University of Medical Science, Semnan, Iran.; ^3^Nervous System Stem Cells Research Center, Semnan University of Medical Sciences, Semnan, Iran.; ^4^Department of Pathology, Semnan University of Medical Sciences, Semnan, Iran.

**Keywords:** Apoptosis, Exercise, Neonatal, Oogenesis, Ovary, Rat.

## Abstract

**Background:**

Regular maternal exercise in pregnancy enhances the physiological, metabolic, and psychological health of mother and fetus.

**Objective:**

To determine the effect of maternal aerobic running during mid or late gestation on plasma levels of estrogen and progesterone and the histological alterations in the ovary of neonatal rats.

**Materials and Methods:**

Twenty-one female Wistar rats were randomly divided into experimental groups to exercises during the 2${}^{\mathrm{nd}}$ or 3${}^{\mathrm{rd}}$ wk of pregnancy (n = 14) and a control group (n = 7). After birth, the neonate's blood was obtained and the estrogen and progesterone levels were evaluated. The ovaries were then removed and used for histological investigations and apoptic assessment.

**Results:**

Higher concentrations of estrogen and progesterone were found in the neonates of the experimental groups (p = 0.001) compared to the control group. The experimental groups had a large ovarian diameter (2${}^{\mathrm{nd}}$ wk: p = 0.044; 3${}^{\mathrm{rd}}$ wk: p = 0.005) and angiogenesis (2${}^{\mathrm{nd}}$ wk: p = 0.003; 3${}^{\mathrm{rd}}$ wk: p = 0.001). In addition, significant enhancements were seen in the the experimental groups in terms of the number (2${}^{\mathrm{nd}}$ wk: p = 0.017; p = 0.035) and diameter (2${}^{\mathrm{nd}}$ wk: p = 0.046; 3${}^{\mathrm{rd}}$ wk: p = 0.004) of primordial follicles, as well as in the diameter of primary oocytes (2${}^{\mathrm{nd}}$ wk: p = 0.073; 3${}^{\mathrm{rd}}$ wk: p = 0.019) compared to the control group. Moreover, rats that exercised had a lower number of apoptotic primordial follicles than the control group (2${}^{\mathrm{nd}}$ wk: p = 0.001; 3${}^{\mathrm{rd}}$ wk: p = 0.001).

**Conclusion:**

It was shown that maternal aerobic running can lead to increased plasma levels of estrogen and progesterone, also improved histological characteristics of the ovary in neonatal rats.

## 1. Introduction

The two main stages in the development of female gonads are known as oocyte formation (oogenesis) and follicle formation (folliculogenesis). Primordial germ cells (PGCs) in rat fetus appear on postcoital (p.c.) day 10, and are mainly established on p.c. day 11. Afterward, at day 12 p.c., almost all of the PGCs (94%) enter the further genital ridges and intrude into the developed genital ridges on p.c. day 13 (1). The differentiation of PGCs into oogonia begins shortly after their arrival in the ovary. Subsequently, the oogonia give rise to oocytes. An oocyte, along with its surrounding flat epithelial cells, is known as a primordial follicle. Accordingly, as development continues, the proliferation of premeiotic oogonia and the apoptosis of oogonia and germ cells happen at different stages of folliculogenesis throughout intrauterine life, and during this process, the follicular reserve is formed (2). For many years, the issue of the ovarian reserve has been considered due to the increased age of marriage as well as the delayed time of first pregnancy.

Exercise has been shown to be important for women's health. In this regard, some clinical and experimental studies have demonstrated that many kinds of sports such as jogging, walking, swimming, and cycling can be performed during pregnancy and are not only without any risks, but also promote the health of the mother and child. Regular maternal physical activates during pregnancy enhance physiological, metabolic, and psychological health and reduces the risk of stillbirth, neonatal deaths, and fetal fat mass (3). Probing the effects of maternal exercise in gestation on the developmental programming of pups during intrauterine and postnatal life is a novel and important research field (4). Accordingly, some studies have indicated that doing exercise during gestation enhances maturation (5), the function of the neonatal brain, and cognition throughout life (6). In this regard, only one study has found a positive effect of maternal physical activity on porcine ovarian development in embryonic, neonatal, and adults (7). Some studies have demonstrated that exercise improves plasma estradiol, progesterone, follicle-stimulating hormone (FSH) (8), ovarian weight, percentage of its proliferating cells (9), and ovary angiogenesis in adult animals (10). Besides, maternal nutrition, health, treatment with estradiol, luteinizing hormone (LH), and FSH during gestation affect the number of oogonia follicles, the percentage of oocytes, and the decreased degeneration of germ cells in the neonate (7, 11, 12).

Several clinical and experimental studies have demonstrated that ovarian follicular reserve, ovulation rates, and age of menarche onset are affected by the intrauterine environment. However, the apecific mechanisms of these changes are still unclear (13). Therefore, in this study, we hypothesized that aerobic running could indirectly affect oogenesis and folliculogenesis in the ovary of neonatal rats through changes in hormones and/or developmental programming. Hence, the purpose of the present study was to evaluate the effects of maternal voluntary aerobic running during mid or late gestation on rat neonatal estrogen and progesterone plasma concentration; ovarian development and its angiogenesis; and development of the primary oocyte, primordial follicle, and their apoptosis.

## 2. Materials and Methods

In this experimental interventional study, 21 (8-wk-old, weighing 200-230 gr) virgin female Wistar rats (*Ratus norvegicus*) were taken from the Central Animal Unit of the Semnan University of Medical Sciences, Semnan, Iran. Two female rats were then caged with male virgin Wistar rats (210 ± 10 gr) to mate for a 24-hr period. Afterward, female rats were evaluated for the existence of a vaginal plug once at midnight and once at 5 am the following day.

Pregnant rats were randomly allocated either into one of the two experimental groups (n = 14) of exercise between 8 and 15 days of pregnancy (2${}^{\mathrm{nd}}$ wk, n = 7) or exercise between 15 and 21 days of pregnancy (3${}^{\mathrm{rd}}$ wk, n = 7) or into the control group (n = 7). Rats were then placed individually in cages in a 12-hr light/dark cycle at 22-24°C, with free access to food and water. The exercise program was similar in both experimental subgroups.

### Voluntary exercise pattern

In the exercise groups, each rat had access to a running wheel (diameter = 34.5 cm, width = 9.5 cm) that was embedded in their cage, and during the 2${}^{\mathrm{nd}}$ and 3${}^{\mathrm{rd}}$ wk of pregnancy, it rotated freely under resistance of 100 gr. Each wheel was linked to a counter that recorded its rotations. Pregnant rats in the control group were put in cages without any access to a running wheel. The 2${}^{\mathrm{nd}}$ wk of pregnancy was defined days 8-14 of pregnancy and the 3${}^{\mathrm{rd}}$ wk as days of 15-21 pregnancy. Only those animals that had run at least 500 m were included in this study. Three and four rats were removed from the 2${}^{\mathrm{nd}}$- and 3${}^{\mathrm{rd}}$-wk groups, respectively, because they had run < 500 m. Finally, the rats were replaced in two groups of experimental.

### Histological study

#### Sample preparation

Twenty-one days post mating, pregnant rats were evaluated twice daily at 9 am and 6 pm up to the time of childbirth. The day the rat neonates were first seen was considered as the postnatal day 0. On postnatal day 0, pups of three female rats were randomly obtained from each colony and blood was extracted from their heart, after sacrificed under anesthesia. The blood samples were then centrifuged with 3000 rpm speed for 10 min. The obtained serum specimens were transferred into micro tubes and then maintained at -20°C until the subsequent experimental steps. Estrogen and progesterone levels were measured using the ELISA test (14). The left ovaries of the neonatal rats were dissected, cleaned of fat, and then fixed in a 10% formalin solution. They were paraffin-embedded and were sectioned into 5-μm thickness transverse serial sections by a sliding microtome (Leitz, Germany). Afterward, the sections were deparaffinized by xylene, and then rehydrated using ethanol and decreasing degrees. Thereafter, the tissue samples were stained with H&E. To perform the morphometric analysis, every fifth tissue section was selected, and the number of follicles was counted in the whole section. To measure the diameter, an image analysis program (ImageJⓇ software) was used. While a primordial follicle is defined as a follicle in which the oocyte is surrounded by flattened granulosa cells, a primary follicle is defined as a follicle in which the oocyte is surrounded by a single layer of cuboidal granulosa cells.

#### TUNEL assay

In this study, immunohistochemistry was performed on 5-µm ovarian sections of paraffin-embedded tissues using an in-situ cell death detection kit (POD, Roche, Germany), following the manufacturer's instructions. Diaminobenzidine (Sigma, St. Louis, MO) was also applied as a chromogen. Tissue samples were deparaffinized using xylene and then hydrated in the ethanol with ascending degrees. The obtained specimens were placed in hydrogen peroxide diluted by methanol (a 10% solution).

Next, they were incubated with proteinase K (Roche, Germany, 30 mg/ml) diluted in phosphate-buffered saline (PBS) for 60 min at 37°C in a humid environment. Tissue samples were incubated with terminal deoxynucleotidyl transferase dUTP nick end labeling (TUNEL) solvent diluted with 50 ml of specific solvent for 60 min at 37°C in a humid environment.

Thereafter, the specimens were incubated with 50 ml of solution convert (Convert POD, HRP) for 60 min at 37°C in a humid environment. Moreover, the tissue specimens were incubated using a chromogenic solution diaminobenzidine tetrachloride for 30 min at laboratory temperature and in wet and dark environment. Finally, these specimens were counterstained using hematoxylin for 5 min, dehydrated in ascending alcohols, and rendered transparent with xylene (15).

The slides were mounted for their evaluation by light microscopy (BX51, Japan). The images were captured using a Nikon, Coolpix, s10 light microscope and were then assessed by two blinded observers.

### Ethical considerations

All procedures were performed following the Guide for the Care and Use of Laboratory Animals of SUMS. The study protocol was approved by the Ethics Committee of SUMS (Code: IR.SEMUMS.REC.1399.108).

### Statistical analysis

Statistic computations were calculated using the SPSS software (Statistical Package for the Social Science, version 22.0 SPSS Inc., Chicago, IL, USA). One-way ANOVA was used to analyze the statistical results between the groups. To compare the differences between groups, Tukey's post hoc test was applied. The obtained data were reported as mean ± SD and p < 0.05 was considered as the level of significance.

## 3. Results

### Running distance of the two subgroups of 2${}^{\mathrm{nd}}$ and 3${}^{\mathrm{rd}}$ wk

Table I shows the average voluntary running distance of pregnant rats during the 2${}^{\mathrm{nd}}$ or 3${}^{\mathrm{rd}}$ wk of pregnancy. The voluntary running distance of pregnant rats during the 2${}^{\mathrm{nd}}$ wk of pregnancy was more than in the 3${}^{\mathrm{rd}}$ wk.

### Maternal voluntary exercise and the estrogen and progesterone in the neonatal rats

According to Table II, the maternal exercise during the 2${}^{\mathrm{nd}}$ or 3${}^{\mathrm{rd}}$ wk of pregnancy was associated with significantly higher plasma levels of estrogen in the rat neonates compared to the control group (p = 0.001, for both exercise groups).

In addition, the plasma levels of progesterone were higher in the groups that experienced in the 2${}^{\mathrm{nd}}$ or 3${}^{\mathrm{rd}}$ wk compared to the control group (p = 0.001, for both exercise groups). The plasma concentration of hormones was not statistically different between the two exercise subgroups (p = 0.076). This suggests that maternal exercise during the 2${}^{\mathrm{nd}}$ or 3${}^{\mathrm{rd}}$ wk of pregnancy can significantly increase the plasma levels of estrogen and progesterone in their pups at the time of birth.

### Effects of maternal voluntary exercise on ovarian diameters in the neonatal rats

Table III shows a comparison of the ovarian diameters of the neonatal rats in the experimental subgroups and the control group. The ovarian diameters of the neonatal rats in the experimental subgroups of exercise during the 2${}^{\mathrm{nd}}$ or 3${}^{\mathrm{rd}}$ wk were significantly larger than those of the control group (p = 0.044, p = 0.005, respectively).

Although exercise seemed to have more of an effect on the ovarian diameter when carried out during the 3${}^{\mathrm{rd}}$ rather than the second wk of pregnancy, this difference was not significant (p = 0.195). Thus, the results of this study suggest that maternal exercise during the 2${}^{\mathrm{nd}}$ or 3${}^{\mathrm{rd}}$ wk of pregnancy may increase their pups' ovary size.

### Effects of maternal voluntary exercise on ovarian angiogenesis in the neonatal rats

Maternal voluntary exercise during the 2${}^{\mathrm{nd}}$ or 3${}^{\mathrm{rd}}$ wk of pregnancy was associated with significantly altered angiogenesis of the ovary in both experimental subgroups (Table III). Notably, there was a significant enhancement in the ovarian angiogenesis of neonatal rats in the subgroups of the 2${}^{\mathrm{nd}}$ and 3${}^{\mathrm{rd}}$ wk compared to the control group (p = 0.003, p = 0.001, respectively).

### Effects of maternal voluntary exercise on the histological features of the ovary in the neonatal rats

**Figure 1:**
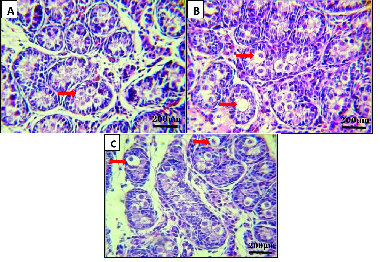
Histological sections of the neonatal rat ovary showing the diameter of primary oocytes in the different groups. Control group (A) maternal exercised during the second wk of pregnancy group (B) and maternal exercise during the third wk of pregnancy group (C). Arrows show a larger diameter of the primary oocytes and their nuclei in the neonatal rat ovary in the maternal exercise groups. Sections were stained with H&E. Magnification is ×400.

As shown in table III, maternal voluntary exercise during the 2${}^{\mathrm{nd}}$ or 3${}^{\mathrm{rd}}$ wk of pregnancy was associated with a significantly higher mean number of primordial follicles compared to the control group (p = 0.017, p = 0.035, respectively). Furthermore, the diameters of the primordial follicles were also significantly larger in the neonatal rats in the experimental 2${}^{\mathrm{nd}}$- and 3${}^{\mathrm{rd}}$-wk exercise subgroups than in the control group (p = 0.046, p = 0.004, respectively). Additionally, the mean diameter of primary oocytes was significantly larger in the neonatal rats with maternal exercise during the 2${}^{\mathrm{nd}}$ or 3${}^{\mathrm{rd}}$ wk of pregnancy compared to the control group (p = 0.073, p = 0.019, respectively). Furthermore, the diameter of the oocyte's nuclei was also significantly larger in the two experimental subgroups in comparison with the control group (p = 0.024, p = 0.003, respectively Figure 1).

**Figure 2:**
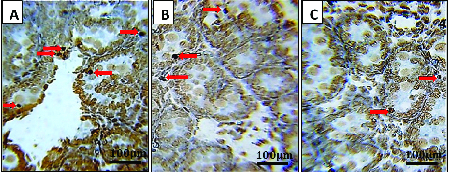
Histological sections of the neonatal rat ovary showing TUNEL-positive cells in the different groups. Immunohistochemistry expression of the apoptotic primordial follicle in the control group (A) the maternal exercise during the second wk of pregnancy group (B) and the maternal exercise during the third wk of pregnancy group (C). Arrows show the apoptotic cells of the ovary in the neonates of the studied groups. Maternal aerobic running during gestation is associated with reduced apoptotic cells compared to the control group. Sections were stained by TUNEL. Magnification is ×400.

A smaller number of atretic primordial follicles was also recorded in the experimental subgroups compared to the control group (p = 0.001, for both exercise groups) (Figure 2).

**Table 1:** Comparison of the total running distance (m/day) of pregnant rats who exercised during the 2${}^{\mathrm{nd}}$ vs. 3${}^{\mathrm{rd}}$ wk of pregnancy


**Days **	**2${}^{\mathrm{nd}}$ wk**	**3${}^{\mathrm{rd}}$ wk**	**d**	**p-value**
**1**	1182.96 ± 527.68	942.14 ± 236.06	0.37	0.001
**2**	1140.28 ± 530.26	884.73 ± 168.51	0.73	0.001
**3**	1259.66 ± 634.99	903.41 ± 142.13	0.91	0.001
**4**	1270.44 ± 752.03	814.2 ± 141.59	1.02	0.001
**5**	1332.94 ± 816.15	737.13 ± 121.58	1.27	0.001
**6**	1284.95 ± 669.07	691.14 ± 101.95	1.54	0.001
**7**	1233.71 ± 592.39	667.69 ± 117.97	1.59	0.001
**Total**	1243.56 ± 620.36	805.8 ± 147.11	1.14	0.001
Data are expressed as Mean ± SEM. Tukey's post hoc test, d: Degree of freedom				

**Table 2:** Effects of maternal voluntary exercise during the 2${}^{\mathrm{nd}}$ or 3${}^{\mathrm{rd}}$ wk of pregnancy on the plasma estrogen and progesterone concentration (ng/ml) of neonatal rats


**Hormones**	**Control**	**2${}^{\mathrm{nd}}$ wk**	**d**	**p-value**	**3${}^{\mathrm{rd}}$ wk**	**d**	**p-value**
**Estrogen **	6.6325 ± 1.35	10.2475 ± 1.5${}^{a}$	2.53	0.001	10.427 ± 1.58${}^{a}$	2.59	0.001
**Progesterone **	0.8975 ± 0.17	1.853 ± 1.42${}^{a}$	0.73	0.001	1.9135 ± 1.61${}^{a}$	0.73	0.001
Data are expressed as Mean ± SD, Tukey's post hoc test. ${}^{a}$P < 0.05 compared with the control group, d: Degree of freedom							

**Table 3:** Effects of maternal voluntary exercise during the 2${}^{\mathrm{nd}}$ or 3${}^{\mathrm{rd}}$ wk of pregnancy on the histological characteristics of the ovary in neonatal rats


<**Histological characteristics of the ovary**		**Control**	**2${}^{\mathrm{nd}}$ wk**	**d**	**p-value**	**3${}^{\mathrm{rd}}$ wk**	**d**	**p-value**
**Ovary**								
	**Diameter (µm)**	495.88 ± 65.58	579.13 ± 51.26${}^{a}$	1.42	0.044	591.44 ± 75.69${}^{a}$	1.35	0.005
	**Angiogenesis (** ***N*** **)**	3.29 ± 1.32	5.17 ± 1.74${}^{a}$	1.22	0.003	6.45 ± 1.93${}^{a}$	1.94	0.001
**Primordial follicle**								
	**N**	18.80 ± 0.38	22.84 ± 2.12${}^{a}$	3.23	0.017	24.01 ± 3.07${}^{a}$	3.02	0.035
	**Diameter (µm)**	27.05 ± 7.25	41.76 ± 9.39${}^{a}$	1.76	0.046	42.86 ± 1.18${}^{a}$	3.75	0.004
	**Atretic (** ***N*** **)**	21.83 ± 6.82	5.8 ± 3.89	-2.99	0.001	5.5 ± 1.47${}^{a}$	-3.94	0.001
**Primary oocyte**								
	**Diameter (µm)**	7.52 ± 0.87	10.15 ± 1.04${}^{a}$	2.75	0.073	10.65 ± 1.11	3.16	0.019
	**Nuclei diameter (µm)**	2.21 ± 0.29	3.22 ± 0.23${}^{a}$	3.88	0.024	3.49 ± 0.6${}^{a}$	2.90	0.003
Data are expressed as Mean ± SD, Tukey's post hoc test. ${}^{a}$P < 0.05 compared with the control group, d: Degree of freedom								

## 4. Discussion

In the current study, the impact of maternal aerobic running during mid or late gestation on the quality of oogenesis and folliculogenesis in the ovary of neonatal rats was examined. Our findings indicated that the plasma estrogen and progesterone concentrations of neonatal rats after birth were considerably higher in the maternal exercise groups compared to the control group. Also, in the exercise during the 2${}^{\mathrm{nd}}$ or 3${}^{\mathrm{rd}}$ wk subgroups, the pups' ovary size, number and diameter of primordial follicles, diameter of primary oocytes and their nuclei, and ovarian angiogenesis were notably higher than those of the control group. In addition, a decreased number of apoptotic primordial follicles was seen in the experimental than in the control group.

Exercise programs are known to be one of the most powerful stimulants of the endocrine system. It has been demonstrated that aerobic exercise can affect leptin levels, and through the hypothalamic-pituitary axis, can also affect estrogen and progesterone concentrations (16). Numerous hormones and growth factors regulate oogenesis and folliculogenesis, including estrogen, which is considered the main regulator (17). Estrogen can control oocyte cyst breakdown and primordial follicle assembly via many pathways in the neonatal mouse ovary (18). Moreover, it can play some roles as an antioxidant, membrane stabilizer, and gene expression regulator (19). There are conflicting reports concerning the impact of maternal exercise on estrogen and progesterone plasma concentrations in neonatal rats. Moradpour reported that exercise can be a physiological stimulus that elevates plasma estrogen and progesterone (20). Also, it has been found that aerobic exercise can reduce body mass index, which causes an increase in estrogen levels (21). The results of these studies are consistent with our results; however, the experimental designs are not similar. Rahnama et al. reported that middle-distance running increased the levels of estradiol in their study, but it had no effect on progesterone, LH, or FSH (22). Besides, it reported that the levels of estradiol, progesterone, LH, and FSH were significantly reduced by doing exercise (23). These inconsistencies may be due to differences in the type, time, duration, and frequency of the exercise.

Our results showed larger ovarian diameters in neonatal rats that had mothers who exercised during pregnancy compared to those that did not, which is in accordance with the results of Kaminski et al. study. Accordingly, they reported increase in the ovarian weight and percentage of proliferating cells after the mothers exercised from mid to late gestation (9). They hypothesized that the increase in umbilical blood flow to the developing fetus may have affected neonatal ovary development. Also, it was demonstrated that moderate exercise increased ovarian arterial blood flow in the mares they studied, leading to improved ovulation (24).

Also, our results showed that angiogenesis was higher in the ovary tissue of neonatal rats whose mothers exercised. Angiogenesis is a complex biological phenomenon that is important for postnatal growth and appropriate embryonic development. Bastu and colleagues in 2018 reported that nitric oxide expression can increase in exercising mice ovaries. Nitric oxide plays an important role in ovarian angiogenesis during folliculogenesis (25). Also, aerobic exercise can elevate markers of expression of angiogenesis (e.g., vascular endothelial growth factor in the subcutaneous adipose tissues of adults) (26). However, it stated that running during pregnancy mitigated Alzheimer-like pathology in the mouse neonates they studied by increasing angiogenesis as well as reducing inflammation (10). Additionally, aerobic training can lead to a significant improvement in serum angiogenic properties in postmenopausal women (27).

It has been previously reported that maternal nutrition and health throughout pregnancy (at the development time of the fetus ovary) can be considered agents that may affect oogonia proliferation and postnatal follicle reserve (7). Several studies have shown that maternal diabetes (28), starvation (18), mobile phone and Wi-Fi exposure (14), and estrogenic compounds (17) during pregnancy have negative effects on primordial follicles pool formation in female rat offspring. In this study, we found that maternal exercise during mid or late pregnancy was associated with higher numbers and diameters of primordial follicles. Furthermore, in the Kaminski's study, it was reported that maternal physical activity during pregnancy can increase the percentage of proliferating cells in porcine embryonic ovaries but not newborn or adult ovaries (9). The number of developing follicles may be increased by obese female mice exercising. It demonstrated that exercise can lead to decrease FOXO1/3 expression in the ovarian follicles, which may affect oocyte count and quality, and the activation of primordial follicles (25). FSH can stimulate the proliferation of ovarian germ cells through the protein kinase B and extracellular regulated protein kinase pathways (11). Also, LH increases the number of follicles with large diameters (27.8-37.5) in the central section and end of the ovarian cortex (12), which is consistent with the results of the current study. Besides, adequate blood flow due to angiogenesis can probably cause the growth of quality oocytes and an increase in the diameters of primordial follicles. A histological study of the rainbow trout ovary following 20-days of swimming observed smaller and less-developed oocytes, which is in contrast to our study results (29). This difference in results may be related to the different animal strains, study design, timing of gestation, and/or different exercise regimens.

Our results also showed that maternal exercise during mid or late pregnancy (2${}^{\mathrm{nd}}$ or 3${}^{\mathrm{rd}}$ wk) was associated with lower apoptosis of primordial follicles. This finding is in accordance with the results of the Qiu and colleagues study, which stated that exercise can decrease atretic follicles in rats with polycystic ovary syndrome (30). Also, it was demonstrated that stress-induced apoptosis in mice liver can be suppressed by voluntary exercise by increasing bcl-2 expression and reducing caspase-3 (31). It has been suggested that exposure to 17alpha-ethynyl estradiol leads to reduced oocyte apoptosis in postnatal ovaries (17). In addition, the positive effects of LH, FSH, and maternal nutrition and health in decreasing the degeneration of germ cells during gestation have also been demonstrated (7, 11, 12).

## 5. Conclusion

According to our results, voluntary aerobic running of pregnant rats during mid or late gestation can increase estrogen and progesterone plasma concentrations, and ovarian size and its angiogenesis in neonates. Furthermore, this type of exercise can increase the primordial follicle/primary oocyte numbers and diameters as well as oocyte nuclei, while decreasing the number of apoptotic primordial follicles. Further studies need to be done to confirm the mechanism of how maternal exercise affects oogenesis and folliculogenesis in the neonatal ovary.

##  Conflict of Interest

The authors declare that there is no conflict of interest in this study.
